# Extended-Spectrum beta (β)-Lactamases and Antibiogram in Enterobacteriaceae from Clinical and Drinking Water Sources from Bahir Dar City, Ethiopia

**DOI:** 10.1371/journal.pone.0166519

**Published:** 2016-11-15

**Authors:** Bayeh Abera, Mulugeta Kibret, Wondemagegn Mulu

**Affiliations:** 1 Department of Microbiology, Immunology and Parasitology, College of Medicine and Health Sciences, Bahir Dar University, Bahir Dar, Ethiopia; 2 Department of Biology, Science College, Bahir Dar University, Bahir Dar, Ethiopia; Australian National University, AUSTRALIA

## Abstract

**Background:**

The spread of Extended-Spectrum beta (β)-Lactamases (ESBL)-producing Enterobacteriaceae has become a serious global problem. ESBL-producing Enterobacteriaceae vary based on differences in antibiotic use, nature of patients and hospital settings. This study was aimed at determining ESBL and antibiogram in Enterobacteriaceae isolates from clinical and drinking water sources in Bahir Dar City, Northwest Ethiopia.

**Methods:**

Enterobacteriaceae species were isolated from clinical materials and tap water using standard culturing procedures from September 2013 to March 2015. ESBL-producing-Enterobacteriaceae were detected using double-disk method by E-test Cefotaxim/cefotaxim+ clavulanic acid and Ceftazidime/ceftazidime+ clavulanic acid (BioMerieux SA, France) on Mueller Hinton agar (Oxoid, UK).

**Results:**

Overall, 274 Enterobacteriaceae were isolated. Of these, 210 (44%) were from patients and 64 (17.1%) were from drinking water. The median age of the patients was 28 years. Urinary tract infection and blood stream infection accounted for 60% and 21.9% of Enterobacteriaceae isolates, respectively. *Klebsiella pneumoniae* was isolated from 9 (75%) of neonatal sepsis. The overall prevalence of ESBL-producing Enterobacteriaceae in clinical and drinking water samples were 57.6% and 9.4%, respectively. The predominant ESBL-producers were *K*. *pneumoniae* 34 (69.4%) and *Escherichia coli* 71 (58.2%). Statistically significant associations were noted between ESBL-producing and non- producing Enterobacteriaceae with regard to age of patients, infected body sites and patient settings (P = 0.001). ESBL-producing Enterobacteriaceae showed higher levels of resistance against chloramphenicol, ciprofloxacin and cotrimoxazole than non-ESBL producers (P = 0.001)

**Conclusions:**

ESBL-producing Enterobacteriaceae coupled with high levels of other antimicrobials become a major concern for treatment of patients with invasive infections such as blood stream infections, neonatal sepsis and urinary tract infections. ESBL-producing Enterobacteriaceae were also detected in drinking water sources.

## Introduction

Extended spectrum β-lactamases (ESBLs) are group of enzymes produced by certain bacteria that degrade broad-spectrum β-lactam antibiotics such as third generation cephalosporins, penicillins and monobactams [1]. ESBLs are often plasmid mediated thereby responsible for rapid transmission of ESBL encoding genes among bacteria. β-lactam antibiotics are widely used for the treatment of pneumonia, urinary tract and bloodstream infections. For instance, a study in Ethiopia stated that amplicillin and ceftriaxone were frequently prescribed in surgical-patients [2].

Enterobacteriaceae includes bacterial species causing infections in the central nervous system, lower respiratory tract, bloodstream, wound and urinary tract sites. ESBL-producing Enterobacteriaceae have been recognized as major multidrug resistant bacteria causing serious infections in hospital acquired and community infections worldwide [3–5].

Several studies showed that the prevalence of ESBL producing Enterobacteriaceae varies with respect to geographical difference, countries, type of hospital settings, nature of patients and antimicrobial prescription patterns [6–9]. In Uganda, 62% of Enterobacteriaceae from clinical isolates were ESBL producer [10]. A study conducted in Gondar, Ethiopia reported that 100% of Enterobacteriaceae from urinary tract infections were ESBL producers [11]. However, other studies in different parts of Ethiopia reported that 36% of *E*. *coli* and 33.3% of *Klebsiella* spp., were ESBL producers [12, 13].

ESBL-Enterobacteriaceae have been detected in different ecological niches in the community and the environment. For instance, ESBL and New Delhi Metallo bata-lactamase 1 (NDM-1) were detected in retail meat and drinking water, respectively [6, 7]. In Bangladesh, 50% *E*. *coli* from river water produce ESBL [14].

The prevailing data on ESBL-producing Enterobacteriaceae has paramount importance for proper treatment of patients. Furthermore, detection of ESBL-producing Enterobacteriaceae from water sources is also important to understand the transmission of ESBL-producing Enterobacteriaceae into human.

Currently, in Ethiopia there is no regular drug resistance surveillance program. Thus, little is known on ESBL-producing Enterobacteriaceae from clinical and drinking water samples. This study was therefore conducted to determine the prevalence of ESBL-producing Enterobacteriaceae in clinical isolates at Felege Hiwot Referral Hospital and water isolates from drinking water sources where the referral hospital is situated (Bahir Dar city), Northwest Ethiopia. Antimicrobial resistance levels of Enterobacteriaceae against commonly prescribed antimicrobials were also determined.

## Materials and Methods

A cross-sectional study was conducted from September 2013 to March 2015. The laboratory investigation was carried out at Bahir Dar Regional Health Research Laboratory Centre which provides state- of-the-art laboratory services to Felege Hiwot Referral Hospital, nearby health centres, private hospitals and clinics in Bahir Dar city, the capital of Amhara Region, Ethiopia.

### Study population

Inpatients and outpatients with clinical diagnosis of bacterial infection from Felege Hiwot Referral Hospital, nearby health centres and private hospitals participated in the study.

#### Sample size and sampling

The sample size for total isolates of Enterobacteriaceae was determined using Epi info version 3.5.1 (public domain software http://www.cdc.gov) considering 95% confidence level, marginal error (5%) and prevalence of 36% ESBL in *E*. *coli* [12]. The sample size was estimated to be 274 Enterobacteriaceae isolates from clinical and water samples. Purposive consecutive sampling method was used to include patients until the required sample size was fulfilled. Patients who were under antimicrobial treatment for duration of two weeks were excluded from the study.

#### Data collection

According to the standard operational procedures for microbial sample collection methods [15], the following specimens were collected: blood, urine, pus, ear discharges, wound swab from surgical site infections and cerebrospinal fluid (CSF). During specimen collection, data such as sex, age, duration of hospital stay and hospital settings were collected using a structured questionnaires.

### Water sample collection

Water samples were collected from household private taps in Bahir Dar City. A total of 280 private tap water samples were collected. A simple random sampling technique using house numbers was used to select water sampling points (private tap water) at households. According to the WHO guideline 200millilitre water samples were collected using sterile glass bottles in the morning [16]. The samples were quickly transported, using a cold chain box to the Microbiology Laboratory at Bahir Dar Regional Health Research Laboratory Center. Water samples were analyzed within 6 hours of collection.

#### Isolation of Enterobacteriaceae

From each specimen, samples were inoculated onto MacConkey agar, Xylose lysine deoxycholate (XLD) agar, Cystine lactose electrolyte deficient agar (CLED), sheep blood agar, and chocolate agar (Oxoid, UK). The plates were incubated at 37°C aerobically for 24 hours [15].

For blood culture, blood samples were aseptically inoculated to Tryptic Soy Broth (Oxoid UK) in 1:5–1:10 proportions and incubated at 37°C. Overnight incubated culture bottles were sub- cultured onto sheep blood, chocolate and MacConkey agar. Culture tubes with negative results were examined for growth daily until fifth day and subsequently subculture when growth was observed. Species of Enterobacteriaceae were identified from pure culture colonies, based on cellular morphologies and biochemical tests as per the standards of microbiology procedure [15].

Isolation and identification of Enterobacteriaceae species from water samples was done using multiple tube fermentation method [15, 17]. Phenotypic bacterial identification of Enterobacteriaceae species was made manually using the following culture media: Urea 40% broth, Simmons citrate agar, sulphide in dole motility medium, Triple Sugar Iron Agar and Klingler Iron Agar and Oxidase test reagents (Oxoid, UK) [17].

### Detection of ESBL-producing Enterobacteriaceae

Detection of phenotypic ESBL-producing Enterobacteriaceae was performed using Double Disc Synregy Test using E-test Cefotaxim/cefotaxim+ clavulanic acid and Ceftazidime/ceftazidime+ clavulanic acid (BioMerieux SA, France) on Mueller Hinton agar (Oxoid, UK). According to the manufacturer’s instructions, ESBL-producing Enterobacteriaceae were confirmed by minimum inhibitory concentration (MIC) ratio of CT/CTL or TZ/TZL. Therefore, where CT ≥ 0.5 and CT/CTL ≥ 8 or TZ ≥ 1and TZ/TZL ≥ 8, it was interpreted as ESBL positive. Furthermore, the presence of a phantom zone or ellipse deformation of the CT or TZ due to mutation was/is deemed ESBL positive [18].

#### Definition

Prevalence of Extended-Spectrum beta (β)-Lactamases (ESBL) in Enterobacteriaceae was defined as this formula:

$$\text{Pr}evalence\;of\;ESBL\equiv \frac{Number\;of\;ESBL-producing\;Enterobacteriaceae}{Total\;Enterobacteriaceae\;isolates}\times 100$$

#### Antimicrobial susceptibility testing

Antimicrobial susceptibility testing was performed by Kirby-Bauer’s disk diffusion method on Mueller Hinton agar (Oxoid, UK). Antimicrobials tested were ciprofloxacin (5μg), trimethoprim /sulphamethoxazole (1.25/23.75μg), ceftriaxone (30μg), and chloramphenicol (30 μg). Susceptibility and resistance were determined according to the guidelines of the Clinical and Laboratory Standards Institute (CLSI) [18].

#### Quality control

Standard strain (*E*. *coli* ATCC 25922^™^) was used to check for E-test for phenotypic ESBL tastings. Moreover, *E*. *coli* (ATCC 25922) and *P*. *aeruginosa* (ATCC 27853) were used to check the potency of antimicrobial discs and to control drug susceptibility testing procedures.

#### Ethics considerations

The Research Ethics Review Committee of the College of Medicine and Health Sciences of Bahir Dar University has approved the ethical clearance. Written consent was obtained from each study participants to provide clinical specimen for diagnosis and research purpose. For children and minors, written consent was obtained from parents and guardian. The Amhara Regional State Health Bureau provided permission to collect drinking water sample from Bahir Dar City. Therefore, the institutional research ethics review committee of the Amhara Regional State Health Bureau, Research and Technology Transfer Core Process approved ethical clearance to do microbial analyse on drinking water samples. Furthermore, each household gave informed consent to allow tap water samples for microbial analysis.

#### Data analysis

Data were analyzed using Statistical package for Social Sciences version 20 Software (*IBM Corp*. *Released 2011*. *IBM SPSS Statistics for Windows*, *Version 20*.*0*. *Armonk*, *NY*: *IBM Corp*). The Chi-square test was employed to compare the association of categorical variables. P-value of less than 0.05 was considered as statistically significant.

## Results

### Patient characteristics

Overall, 477 patients took part in this study. Enterobacteriaceae were isolated from 210 (44%) patients). Among 210 patients with Enterobacteriaceae infections, the majority were females (65.7%). The median age of the patients was 28 years. Children under 5 years of age accounted for 58 (27.62%) of the cases and 12 (20.7%) were neonates less than 28 days old. Among Enterobacteriaceae isolates, 34.3% were from inpatients. The majority of the Enterobacteriaceae were isolated from urinary tract and blood stream infections (Table 1).

**Table 1:** Characteristic of patients with Enterobacteriaceaee isolates, N (%).

Variables	*E*. *coli*	*K*. *pneumoniae*	*P*. *mirabilis*	*E*. *cloacae*	*Citrobacter* spp.
**Sex**					
Female (n = 128)	78 (61.0)	28 (21.9)	18 (14.1)	2 (1.6)	2 (1.6)
Male (n = 82)	44 (53.6)	21 (25.6)	11(13.4)	6 (7.3)	-
**Age (years)**					
0< 5 (n = 58)	24 (41.4)	26 (44.8)	4 (6.9)	2 (3.4)	2 (3.4)
6–20 (n = 20)	14 (70)	-	5 (25)	1 (5)	-
≥ 21 (n = 132)	84 (63.6)	23 (17.4)	20 (15.20	5 (3.8)	-
**Patient settings**					
Inpatients (n = 72)	32 (44.4)	33 (45.8)	2 (2.7)	3 (4.1)	2 (1.4)
Outpatients (n = 138)	90 (65.2)	16 (11.6)	27 (19.5)	5 (3.6)	
**Clinical samples**					
Urine (n = 126)	84 (66.6)	21(16.6)	17 (13.5)	4 (3.2)	-
Blood (n = 46)	15 (32.6)	26 (56.5)	-	3 (6.5)	2 (4.3)
Open wounds (n = 22)	17 (77.2)	-	4 (18.2)	1 (4.5)	-
Ear discharge (n = 8)	-	-	8 (100)	-	-
CSF (n = 8)	6 (75)	2 (25)	-	-	-
**Total**	122 (58.1)	49 (23.3)	29 (13.8)	8 (3.8)	2 (0.9)

As shown in Table 1, *E*. *coli* was the most commonly isolated organism (58.1%), followed by *K*. *pneumoniae* (23.3%). An important observation in this study is that 44.8% of *K*. *pneumoniae* was isolated from children under 5 years of age. Of these, *K*. *pneumoniae* accounted for 9 (75%) neonatal sepsis. Enterobacteriaceae species were isolated from 48 drinking tap water samples (17.1%). A total of 48 *E*. *coli* and six *K*. *pneumoniae* were isolated in 280 drinking tap water samples.

### ESBL-producing Enterobacteriaceae

Table 2 depicts that the overall prevalence of ESBL-producing Enterobacteriaceae in clinical and drinking water samples. The prevalence of ESBL-producing Enterobacteriaceae in clinical and drinking water samples were 57.6% and 9.4%, respectively. *K*. *pneumoniae* and *E*. *coli* were the major ESBL-producer Enterobacteriaceae species in clinical samples.

**Table 2:** Prevalence of ESBL-producing Enterobacteriaceae species in clinical and water samples.

Enterobacteriaceae species	Prevalence of ESBL	
	Clinical isolates	Water isolates
*E*. *coli*	71/122 (58.2)	2/48 (4.2)
*K*. *pneumoniae*	34/49 (69.4)	2/6 (33.3)
*P*. *mirabilis*	6/29 (20.7)	0/6 (00
*E*. *cloacae*	7/8 (87.5)	2/4 (50)
*Citrobacter* spp.	2/2 (100)	-
Total ESBL producer	121/210 (57.1)	6/64 (9.4)

Table 3 compares the ESBL-producing Enterobacteriaceae with patient characteristics. Statistically significant association was noted between ESBL-producing and non-ESBL Enterobacteriaceae with anatomically infected sites (P = 0.001). For instance, from blood and open wound swabs, 84.8% and 72.7% of isolates were ESBL-producer, respectively. Furthermore, statistically significant associations was observed between ESBL-producer and non-ESBL Enterobacteriaceae in regard to age and patient settings (P = 0.001). However, there were no significant association between ESBL-producers and non-ESBL producer and gender of patients and duration of hospital stay.

**Table 3:** Association of ESBL-producing Enterobacteriaceae with patients’ characteristics.

Patients characteristics	ESBL- producer N (%)	Non- ESBL- producer N (%)	P value
Sex			
Female	74 (57.8)	54 (42.2)	0.318
Male	46 (56.1)	36 (43.9)	
Age (years)			
0–5	43 (79.6)	11 (20.4)	0.001
6–20	8 (50)	8 (50)	
≥ 21	61 (49.1)	63 (50.9)	
Patient settings			
Inpatients	58 (80.5)	14 (19.5)	0.001
Outpatients	62 (44.9)	76 (50.1)	
Clinical sample			
Urine	58 (46)	52 (54)	0.001
Blood	39 (84.8)	7 (15.2)	
Others^#^	24 (63.1)	14 (36.9)	
Hospital stay			
2–5 days (n = 40)	32 (80)	8 (20)	0.44
≥ 6 days (n = 34)	30 (88.2)	4 (11.8)	
Total	120 (57.1)	90 (42.9)	

Key:

^#^ include clinical samples from ear discharge, open wound and cerebrospinal fluids *(CSF)*.

### Antibiogram levels

Table 4 shows the antimicrobial resistance profiles of ESBL-producing and non-producing Enterobacteriaceae against commonly prescribed antimicrobials. Overall, ESBL-producing Enterobacteriaceae showed higher levels of resistance against chloramphenicol, ciprofloxacin and cotrimoxazole than non-ESBL producers (P = 0.001). Enterobacteriaceae species from clinical sample revealed 70% and 47.1% of resistance to cotrimoxazole and ciprofloxacin, respectively. All *E*. *cloacae* revealed resistance to chloramphenicol, ciprofloxacin and cotrimoxazole. Overall, high levels of resistance for Enterobacteriaceae were exhibited against cotrimoxazole. *E*. *cloacae* from clinical samples revealed the highest levels of resistance. Although, isolation rate of *K*. *pneumoniae* in drinking water is low, it revealed high levels of resistance to chloramphenicol, ciprofloxacin and cotrimoxazole.

**Table 4:** Comparison of antibiogram profiles of clinical and water isolates; and ESBL-producing and non-producing Enterobacteriaceae.

Enterobacteriacaea species	Chloramphenicol		Ciprofloxacin		Cotrimoxazole	
	R	S	R	S	R	S
**Clinical isolates**						
*E*. *coli* (n = 122)	61 (50)	55 (45.1)	49 (40)	74 (60)	82 (67.2)	40 (32.8)
*K*. *pneumoniae* (n = 49)	25 (51)	24 (49)	28 (57)	21 (43)	30 (61)	19 (39)
*P*. *mirabilis* (n = 29)	13 (44.8)	16 (55.2)	10 (34.4)	20 (65.6)	18 (62)	11 (38)
*E*. *cloacae* (n = 8)	8 (100)	-	2 (100)	-	8 (100)	-
*Citrobacter* spp., (n = 2)	2 (100)	-	-	-	2 (100)	-
Overall (n = 210)	106 (50.4)	104 (49.6)	99 (47)	111 (53)	147 (70)	63 (30)
**Water isolates**						
*E*. *coli* (n = 48)	20 (41.6)	28 (59.4)	3 (8.3)	45 (91.7)	23 (48)	25 (52)
*K*. *pneumoniae* (n = 6)	3 (50)	3 (50)	4 (66.6)	2 (33.4)	4 (66.6)	2 (33.4)
*P*. *mirabilis* (n = 6)	-	6 (100)	-	6 (100)	-	6 (100)
*E*. *cloacae* (n = 4)	-	4 (100)	-	4 (100)	-	4 (100)
Overall (n = 64)	23 (36)	41 (64)	7 (10.9)	57 (89.1)	27 (42.2)	37 (57.8)
**ESBL-producer**	78.1	28.2	57.8	42.2	81.3	18.7
**Non-ESBL producer**	31.7	68.3	19.5	80.5	48.8	51.2
P value	0.001		0.001		0.001	

## Discussion

In the present study, Enterobacteriaceae species were frequently isolated from urinary tract and blood stream infections. *K*. *pneumoniae* accounted for 75% neonatal sepsis. Therefore, prevailing data on ESBL-producing Enterobacteriaceae is essential for proper treatment of invasive infections caused by Enterobacteriaceae species. This study demonstrated simultaneously the prevalence of ESBL-producing Enterobacteriaceae in clinical and drinking tap water samples.

Overall, 57.6% of Enterobacteriaceae isolated from clinical samples were phenotypic ESBL-producers. Likewise, 62% prevalence of ESBL in Enterobacteriaceae was detected in Uganda [10]. However, a study in Ethiopia reported that all Enterobacteriaceae from urinary tract infections were ESBL-positive [11]. In this study, majority of (75–85%) Enterobacteriaceae from blood, CSF and wound isolates were ESBL producer. Therefore, ESBL-producing strains of Enterobacteriaceae pose a big challenge in treatment of patients with invasive bacterial infections.

Statistically significant association was noted between ESBL-producer and patients’ age (P = 0.001). The prevalence of ESBL-producing Enterobacteriaceae in 0–5 years was 79.6%. The reason for this might be due to the fact that *K*. *pneumoniae* was the most frequent (44.8%) causes of infections in children under 5 years of age. However, comparatively low prevalence of ESBL (40%) was reported in paediatric infection in Thailand [19]. Therefore, children infected with Enterobacteriaceae species need a great emphasis prior to selection of antimicrobial therapy. Furthermore, patient settings and types of clinical samples had statistically significant association with ESBL-producing Enterobacteriaceae (P = 0.001). For example, Enterobacteriaceae isolates from inpatients were 80.5% ESBL-producer while 44.9% were from outpatients. However, statistically significant association was not observed between duration of hospitalization and ESBL-producing Enterobacteriaceae.

The prevalence of ESBL-producing *K*. *pneumoniae* and *E*. *coli* in this study was higher than previous studies in Ethiopia. For instance, 33.3% ESBL positive in *K*. *pneumoniae* and 36% of ESBL-positive in *E*. *coli* were reported in Ethiopia [12, 13]. Conversely, 100% ESBL-producers were reported from urinary tract infection in Ethiopia [11]. These variations might be due to difference in methods of ESBL detection and types of patients. Similarly, Kateregga *et al*. (2015) reported 72.7% and 58.1% of ESBL positive in *K*. *pneumoniae* and *E*. *coli*, respectively in Uganda [10]. Furthermore, 77.7% of ESBL-producing *K*. *pneumoniae* was reported in Iran [20].

The present study detected ESBL-producing Enterobacteriaceae from drinking water sources. Of these, *K*. *pneumoniae* was the most ESBL-producer. Spread of ESBL-producing Enterobacteriaceae through drinking water into human gut pose a threat for empiric treatment of invasive infections like urinary tract and blood stream infections. Studies conducted in different parts of Africa detected ESBL-producing Enterobacteriaceae. Studies from Nigeria and Democratic Republic of Congo reported 7.14% and 5.3% prevalence of ESBL-producing Enterobacteriaceae in drinking water sources, respectively [21, 22].

*Escherichia coli* from drinking water exhibited high level resistance to cotrimoxazole (48%) and chloramphenicol (41.6%). Studies from India and Egypt reported that *E*. *coli* showed 53% and 38.5% resistance to cotrimoxazole, respectively [23, 24]. This study therefore strengthens the great concern of multidrug- resistant bacteria dissemination through environmental niches like water sources [25, 26].

ESBL-producing Enterobacteriaceae showed higher levels of resistance to chloramphenicol, ciprofloxacin and cotrimoxazole than non-ESBL producer (P = 0.001). Similarly, studies stated that ESBL-producing Enterobacteriaceae showed high levels of resistance to other antibiotics like cotrimoxazole and ciprofloxacin [26, 27]. Therefore, ESBL-producing strains are multi-drug resistant bacteria thus pose challenging scenario for empirical therapy of invasive infections.

The major limitation of the study was that ESBL detection was only performed phenotypically using Double Disk Synergy Testing by E-test method. The other limitation of this study was that drinking water sources for detection of ESBL were collected from Bahir Dar city not from the hospital pipelines. Therefore, it was difficult to associate ESBL-producing strains of clinical isolates from hospital with that of drinking water sources.

## Conclusions

This study demonstrated high prevalence of ESBL-producing Enterobacteriaceae in hospitalized and outpatients. *K*. *pneumonia*, *E*. *coli* and *E*. *cloaeae* were the major ESBL-producer. Overall, ESBL-producing Enterobacteriaceae exhibited higher levels of resistance than non- ESBL producer for chloramphenicol, ciprofloxacin and cotrimoxazole. The presences of phenotypic ESBL-producing Enterobacteriaceae were detected in drinking water. Screening for ESBL in patients with invasive infections needs to be initiated in the hospital. We recommend further studies on molecular epidemiology of ESBL producing Enterobacteriaceae and their impacts on patients and health care in the area.

## References

[1] Paterson DL, Bonomo RA. Extended-spectrum beta-lactamases: a clinical update. *Clin Microbiol Rev*. 2005; 18:657–686. 10.1128/CMR.18.4.657-686.2005 16223952 PMC1265908

[2] Abula T, Kedir M. The pattern of antibiotic usage in surgical in-patients of a teaching hospital, northwest Ethiopia. *Ethiop J Health Dev*. 2004;18 (1):35–38.

[3] Aruna K, Mobashshera T. Prevalence of Extended spectrum beta-lactamse production among uropathogens in south Mumbai and its antibiogram patterns. *EXCLI Journal*. 2012; 11:363–372. 27418912 PMC4942789

[4] Brolund A. Overview of ESBL-producing Enterobacteriaceae from a Nordic perspective. *Infect Ecol Epidemiol*. 2014; 4: 24555 10.3402/iee.v4.24555. PMC4185132 25317262

[5] Pitout JDD, Nordmann P, Laupland KB, Poirel L. Emergence of Enterobacteriaceae producing extended-spectrum b-lactamases (ESBLs) in the community. *J Antimicrob Chemother*. 2005; 56: 52–59. 10.1093/jac/dki166 15917288

[6] Overdevest I, Willemsen I, Rijnsburger M, Eustace A, Xu L, Hawkey P, et al Extended-spectrum β-lactamase genes of *Escherichia coli* in chicken meat and humans, The Netherlands. *Emerg Infect Dis*. 2011; 17:1216–22. 10.3201/eid1707.110209 21762575 PMC3381403

[7] Walsh TR, Weeks J, Livermore DM, Toleman MA: Dissemination of NDM-1 positive bacteria in the New Delhi environment and its implications for human health: an environmental point prevalence study. *Lancet Infect Dis*. 2011; 11:355–362 10.1016/S1473-3099(11)70059-7 21478057

[8] Johnson JR, Russo TA. Uropathogenic *Escherichia coli* as agent of diverse non-urinary tract extraintestinal infections. *J Infect Dis*. 2002; 186: 859–864. 10.1086/342490 12198625

[9] Pitout JD, Laupland KB. Extended-spectrum beta-lactamase-producing Enterobacteriaceae: an emerging public-health concern. *Lancet Infect Dis*. 2008; 8:159–166. 10.1016/S1473-3099(08)70041-0 18291338

[10] Kateregga JK, Kantume R, Atuhaire C, Lubowa MN, Ndukui JG. Phenotypic expression and prevalence of ESBL-producing Enterobacteriaceae in samples collected from patients in various wards of Mulago Hospital, Uganda. *BMC Pharmacol Toxicol*. 2015;16:14 10.1186/s40360-015-0013-1 26031914 PMC4451872

[11] Eshetie S, Unakal C, Gelaw A, Ayelign B, Endris M, Moges F. Multidrug resistant and carbapenemase producing Enterobacteriaceae among patients with urinary tract infection at refral Hospital, Northwest Ethiopia. *Antimicrob Resist Infect Control*. 2015; 4:12 10,1186. 10.1186/s13756-015-0054-7 25908966 PMC4407313

[12] Mulualem Y, Kasa T, Mekonnen Z, Suleman S. Occurrence of extended spectrum beta (b)-lactamases in multi-drug resistant *Escherichia coli* isolated from a clinical setting in Jimma University Specialized Hospital, Jimma, and Southwest Ethiopia. *East Afr J Public Health*. 2012; 9 (2):58–61. 23139958

[13] Seid J, Asrat D. Occurrence of extended spectrum beta-lactamse enzymes in clinical isolates of *Klebsiella* species from Harar region, eastern Ethiopia. *Acta Trop*. 2005; 95 (2);143–148. 10.1016/j.actatropica.2005.05.009 15993831

[14] Rashid M, Rakib MM, Hasan B. Antimicrobial-resistant and ESBL-producing *Escherichia coli* in different ecological niches in Bangladesh. *Infect EcolEpidemiol*. 2015;5: 26712- 10.3402/iee.v5.26712. PMC4507753 26193990

[15] Cheesbrough M. Manual of medical microbiology. Low price ed Britain: Oxford press; 2000p.251–260.

[16] World Health Organization. Guidelines for Drinking Water Quality, 3rd edn, Recommendations. WHO, 2004, Geneva, pp. 1–540.

[17] World Health Organization. Basic Laboratory Procedures in Clinical Bacteriology, 2^nd^ ed WHO, 2003, Geneva, pp. 37–50.

[18] Clinical Laboratory Standards Institute. Performance standards for antimicrobial susceptibility testing; twenty-first informational supplements. M100-S21. Wayne PA, USA; CLSI, 2013.

[19] Sethaphanich N, Santanirand P, Rattanasiri S, Techasaensiri C, Chaisavaneeyakorn S, Apiwattanakul N, et al Pediatric ESBL infection: Community acquired infection and treatment options. *Pediatr Int*. 2015; 10.1111/ped.12845 26513341

[20] Mehrgan H, Rahbar M, Halvaii ZA. High prevalence of Extended Spectrum BetaLactamase-Producing *Klebsiella pneumoniae* in a tertiary care hospital in Tehran, Iran. *J Infect Dev Ctries*. 2010; 4 (3): 132–138. 20351452 10.3855/jidc.488

[21] Odumosu BT, Akintimehin AR. Occurrence of Extended-Spectrum Beta-Lactamase producing Enterobacteriaceae isolates in communal water sources in Ogun State, Nigeria. *Afr J*. *Clin Exper Microbiol*. 2014; 16 (1): 28–32.

[22] Boeck HD, Miwanda B, Lunguya-Metila O, Muyembe-Tamfum JJ, Stobberingh E, Glupczynski Y, et al ESBL-Positive Enterobacteria Isolates in Drinking Water. *Emerg Infect Dis*. 2012; 18 (6): 1019–1020. 10.3201/eid1806.111214 22608263 PMC3358152

[23] Sahoo KC, Tamhankar AJ, Sahoo S, Sahu SP, Klintz SR, Lundborg CS. Geographical variation in antibioticresistant *Escherichia coli* isolates from stool, cow-dung and drinking water. *Int J Environ Res Public Health*. 2012; 9:746–759. 10.3390/ijerph9030746 22690160 PMC3367274

[24] Alzahrani A M, Gherbawy YA. Antibiotic resistance in *Escherichia coli* strains isolated from water springs in Al- Ahsa Region. *Afr J Microbiol Res*. 2011; 5:123–130.

[25] Abera B, Kibret M, Goshu G, Yimer M. Bacterial quality of drinking water sources and antimicrobial resistance profile of Enterobacteriaceae in Bahir Dar city, Ethiopia. *J Water Sanit Hygi Dev*. 2014; 4: 384–390.

[26] Blaak H, Lynch G, Italiaander R, Hamidjaja RA, Schets FM, de Roda Husman AM. Multidrug-Resistant and Extended Spectrum BetaLactamase-Producing *Escherichia coli* in Dutch Surface Water and Wastewater. *PLoS ONE*. 2015; 10(6): e0127752 10.1371/journal.pone.0127752 26030904 PMC4452230

[27] Somily AM, Habib HA, Absar MM, Arshad MZ, Manneh K, Subaie SS, et al ESBL-producing *Escherichia coli* and *Klebsiella pneumoniae* at a tertiary care hospital in Saudi Arabia. *J Infect Dev Ctries*. 2014; 8 (9): 1129–1136. 10.3855/jidc.4292 25212077

